# Experimental Study on the Inhibition of RANKL-Induced Osteoclast Differentiation In Vitro by Metformin Hydrochloride

**DOI:** 10.1155/2022/6778332

**Published:** 2022-09-12

**Authors:** Zhang Chen, Liu Zige, Yeow Sai Kiang, Chen Desheng

**Affiliations:** ^1^Department of Orthopedic Surgery, General Hospital of Ningxia Medical University, Yinchuan, China; ^2^School of Clinical Medicine, Guangxi Medical University, Nanning, China; ^3^Department of Orthopedic Surgery, Sengkang General Hospital, Singapore; ^4^Department of Orthopedic Surgery, People's Hospital of Ningxia Hui Autonomous Region, Yinchuan, China

## Abstract

**Objective:**

Establishment of an in vitro osteoclast induction model under nuclear factor-*κ*B receptor activator ligand (RANKL) induction for investigating the effect of metformin hydrochloride (Met) on osteoclast differentiation.

**Methods:**

RANKL induced the differentiation of mouse bone marrow macrophages (BMMs) into osteoclasts in vitro, and Met was added at different concentrations for intervention during the induction process. After 5 d of culture and fixation, the number of osteoclasts was counted by tartrate-resistant acid phosphatase (TRAP) staining and F-actin staining, and the function of osteoclasts was examined with hydroxyapatite-coated plates. Real-time fluorescence quantitative PCR was performed to detect the expression of *Cathepsin K*, osteoclast associated receptor (*OSCAR*), and *TRAP*, and the effect of Met on Mitogen-activated protein kinases (MAPK) signaling pathway was detected by Western blot.

**Results:**

Met significantly reduced osteoclast formation, F-actin ring formation, bone resorption, and the expression of relevant genes *Cathepsin K*, *OSCAR*, and *TRAP*. The Western blotting study demonstrated that Met inhibited the MAPK signaling pathway by decreasing the phosphorylation of extracellular regulated protein kinase (ERK), which plays important roles in osteoclast formation.

**Conclusion:**

Metformin hydrochloride inhibited the differentiation of osteoclasts, decreased the bone resorption area, and suppressed phosphorylation of ERK in vitro.

## 1. Introduction

Bone reconstruction in the human body includes two mutually balanced processes of bone formation and bone resorption, which are regulated by osteoblasts and osteoclasts, respectively [1]. Excessive activation of osteoclasts leads to increased bone resorption and disrupts the balance between bone formation and bone resorption, which will cause diseases such as osteoporosis, in which the role of osteoclasts is crucial [2]. M-CSF and RANKL play a vital role in osteoclastogenesis in the human body. The main role of M-CSF is to promote the proliferation and survival of osteoclast precursor cells, also known as mononuclear macrophages; RANKL, on the other hand, is secreted by osteoblasts and triggers the differentiation of osteoclastic precursor cells into osteoclasts as an important factor [3].

**Figure 1:**
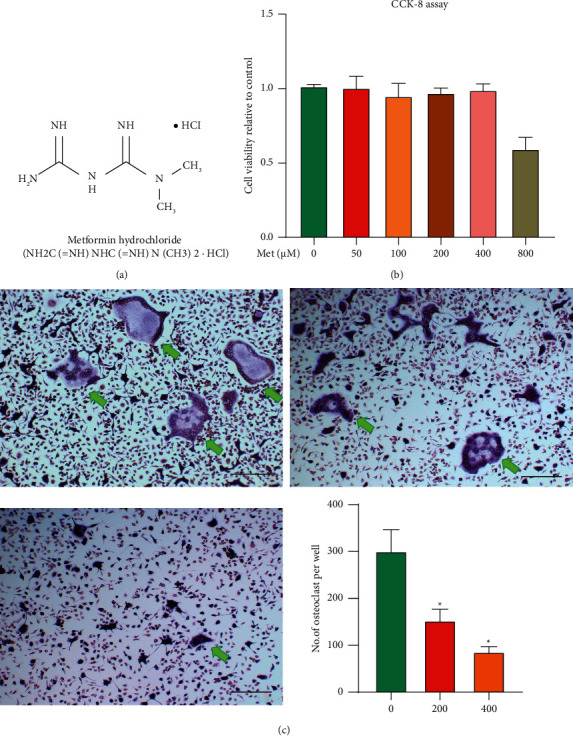
Met reduces RANKL-induced osteoclastogenesis by no cytotoxicity. (a) Chemical structure of Met. (b) The viability of cells unaffected by Met treatment at a concentration of 400 *μ*M. (c) BMMs stimulated with specified concentrations of Met in osteoclastogenesis media (0, 200, and 400 *μ*M), fixed, and stained with TRAP (^*∗*^*p* < 0.05, scar bar = 100 *μ*m). The green arrows indicate TRAP-positive cells.

Currently, it has been found that all types of diabetes increase the risk of osteoporosis [4]. The use of glucose-lowering drugs to reduce blood glucose can significantly reduce the risk of fracture, and of these glucose-lowering drugs, Met (Figure 1(a)) reduces the risk of fracture most significantly [5]. Current studies have shown that Met increases bone mineral density (BMD) by promoting osteoblast differentiation [6–8] and enhancing the function of osteoblasts. With the further study of Met, researchers found that in addition to its hypoglycemic effect, Met can promote the differentiation of bone marrow mesenchymal stem cells to osteoblasts, increase the number of osteoblasts, and promote bone formation. However, the specific mechanism of the effect of Met on bone metabolism is still not fully understood, and the effect of metformin on osteoclasts is rarely reported in the literature. Therefore, this study aims to explore the effect of Met on osteoclast differentiation and function, which may provide a potential treatment for osteoporosis.

## 2. Materials and Methods

### 2.1. Cell Culture and CCK-8 Cell Viability Assay

BMMs were isolated as previously described by our work [2]. Briefly, the mice were sacrificed and sterilized. Bilateral femurs and tibias were separated in the ultraclean bench, placed in prechilled sterile PBS, and the ends of the bone tissue were removed with scissors to fully expose the bone marrow cavity. The bone marrow cavity is repeatedly flushed with the prepared medium 2-3 times.

Centrifuge the rinse solution to collect the cells and then incubate in Dulbecco's Modified Eagle Medium (DMEM; Gibco, Thermo Fisher Scientific, Inc., Waltham, MA, USA) containing 10% fetal bovine serum (FBS; Gibco, Thermo Fisher Scientific, Inc.), 100 IU/mL penicillin, 100 *µ*g/mL streptomycin (Beijing Solarbio Science and Technology Co., Ltd., Beijing, China), and 50 ng/mL of macrophage colony-stimulating factor (M-CSF; R&D Systems, Minneapolis, MN, USA). When the cells were grown to 90% fusion, the adnexal cells were gently scraped off with a cell scraper and these were considered to be BMMs.

To evaluate the Met-dose used in this research without toxic effects on BMMs, a cell viability assay (cell counting kit-8 (CCK-8); Jiangsu KeyGen Biotech Co., Ltd., Nanjing, China) was performed. BMMs (5 × 10^3^ cells/well) were seeded in a 96-well plate and incubated with M-CSF (50 ng/mL) and RANKL (100 ng/mL, R&D Systems, Minneapolis, MN, USA). Subsequently, cells were treated with the indicated doses of Met (0, 50, 100, 200, 400, and 800 *μ*mol/L, Sigma, St. Louis, MO, USA) for 72 h. Then, the culture solution in each well was discarded and DMEM culture solution (without FBS) containing 10 *μ*g CCK-8 assay solution was added and incubated for 2 h. The absorbance at 450 nm was then measured by a microplate reader (Thermo Scientific, Waltham, Massachusetts, USA), charted, and statistically analyzed.

### 2.2. TRAP and F-Actin Ring Staining Assay

BMMs were induced to become mature osteoclasts by RANKL in 96-well plates with 8000 cells per well containing 2 mL of complete medium, 50 ng/mL M-CSF, and 100 ng/mL RANKL with different concentrations of Met (200 and 400 *μ*M), and the culture medium was changed every 2 d for a total of 5 d.

TRAP: After 5 d, the cells were fixed for 30 min and stained with antitartrate acid phosphatase dye for 15 min, and then, the number of osteoclasts was counted under a 20 × microscope (osteoclasts with nuclei greater than 3 were counted).

F-actin: After 5 d, the cells were fixed for 10–15 min. The fixative was discarded, and the cell was rinsed with PBS and permeabilized with 0.1% (v/v) Triton X-100 for 5 min. Subsequently add 1 : 100 dilution of Alexa-Fluor 647 phalloidin (Invitrogen; San Diego, CA, USA) and incubate for 1 h at room temperature. Then, BMMs were rinsed three times with PBS, and the nuclei were counterstained with DAPI. Photograph and observe the stained cells under a confocal microscope (Olympus, Tokyo, Japan).

### 2.3. Resorption Pit Formation Assay

BMMs were induced to become osteoclasts by RANKL in a 6-well plate, and the cells were obtained by digestion and seeded on a bone resorption plate covered with hydroxy-limestone (Corning Inc., NY, USA). 2.4 × 10^4^ cells per well containing 2 mL of complete medium, 50 ng/mL M-CSF, and 100 ng/mL RANKL were added at different concentrations of Met (200 and 400 *μ*M). After 5 d of incubation, the cells were washed off the plates with 10% bleach solution, and then, the bone resorption traps were photographed with a microscope and the bone resorption area was calculated by Image J (NIH, Bethesda, MD, USA).

### 2.4. Gene Expression of *Cathepsin K*, Calcitonin Receptor, and *TRAP*

Osteoclast-related genes were detected by real-time fluorescence quantitative PCR. BMMs were inoculated in 6-well plates with 2.4 × 10^4^ cells per well, containing 2 mL complete medium, 50 ng/mL M-CSF, and 100 ng/mL RANKL with different concentrations of Met (200 and 400 *μ*M). The solution was changed every other day and cultured for 5 d. We used TRIzol reagent (Sigma-Aldrich, St. Louis, MO, USA) to extract total ribonucleic acid (RNA) from the cells, which was followed by RNA reverse transcription using the Revert Aid First Strand cDNA Synthesis kit (Thermo Fisher) according to the manufacturer's instructions and amplification using a suitable primer as follows: *Cathepsin K* (forward 5′-GGGAGAAAAACCTGAAG-3′ and reverse: 5′-ATTCTGGGGACTCAGAGAGC-3′); *OSCAR* (forward: 5′- CTGCTGGTAACGGATCAGCTCCCCAGA -3′ and reverse 5′-CCAAGGAGCCAGAACCTTCGAAACT-3′); *TRAP* (forward 5′-TGTGGCCATCTTTATGCT-3′ and reverse 5′-GTCATTTCTTTGGGGCTT-3′); and *GAPDH* (forward: 5′-CCTCGTCCCGTAGACAAAATG-3′ and reverse 5′-TGAGGTCAATGAAGGGGTCGT-3′). *GAPDH* was used as an internal reference to determine the messenger RNA (mRNA) levels.

### 2.5. Western Blot Assay

BMMs were inoculated in 6-well plates with 2.4 × 10^4^ cells per well, containing 2 mL of complete medium, 50 ng/mL M-CSF, and 100 ng/mL RANKL, and different concentrations of metformin hydrochloride (200 and 400 *μ*M) were added. The medium was changed every other day and discarded after 5 d of incubation. Cells were lysed by shaking in radioimmunoprecipitation assay (RIPA) lysis buffer supplemented with protease inhibitor (Jiangsu KeyGen Biotech Co., Ltd., Nanjing, China). The bicinchoninic acid (BCA) assay (Jiangsu KeyGen Biotech) measured the protein concentration. Cell extract (30 *μ*g) was separated using 10% sodium dodecyl sulfate (SDS)-polyacrylamide gel electrophoresis and transferred to polyvinylidene fluoride (PVDF) membranes; 5% skim milk dissolved in Tris-buffered saline (TBS)-Tween (10 mM Tris-HCl, 50 mM NaCl, 0.25% Tween 20) was used to block the membranes for 1 h. The membrane was incubated overnight at 4°C with primary antibodies (ERK, 1 : 1 000; p-ERK, 1 : 700; p38, 1 : 1 000; p-p38, 1 : 700; JNK, 1 : 1 000; p-JNK, 1 : 800; *β*-actin, 1 : 2 000; Cell Signaling Technology).

On the next day, the membrane was washed three times with PBST and incubated with HRP-antirabbit secondary antibody (1 : 5000; Proteintech) for 1 h. The bands were visualized by a BIO-RAD imaging system (Bio-Rad Laboratories, Inc. Hercules, CA, USA). *β*-Actin was used as the internal reference.

### 2.6. Statistical Analysis

Measured data were analyzed using analysis of variance (ANOVA). The difference between the two groups was analyzed using Student's *t*-test. All statistical analyses were performed using the SPSS 22.0 (IBM Corp., Armonk, NY, USA), and ^*∗*^*p* < 0.05 was considered significant.

## 3. Results

### 3.1. Met Suppresses the Production of Osteoclasts without Cytotoxicity

BMMs were cultured to see how Met affected osteoclastogenesis. We used a CCK-8 test to determine Met's cytotoxicity before confirming the safe dose. Met did not affect BMMs viability at concentrations less than 400 *μ*M, according to the results (Figure 1(b)).

Then, for 5 days, RANKL and M-CSF-induced BMMs were treated with different doses of Met (200 and 400 *μ*M), and TRAP and F-actin tests were performed to see how Met affected osteoclastogenesis. Met reduced RANKL-stimulated osteoclastogenesis in a dose-dependent manner, as evidenced by the considerably smaller area of TRAP-positive osteoclasts and F-actin ring in Met-treated groups compared to Sham-treated groups (Figure 1(c)).

### 3.2. Effect of Met on the Bone Resorption Capacity of Osteoclasts

**Figure 2:**
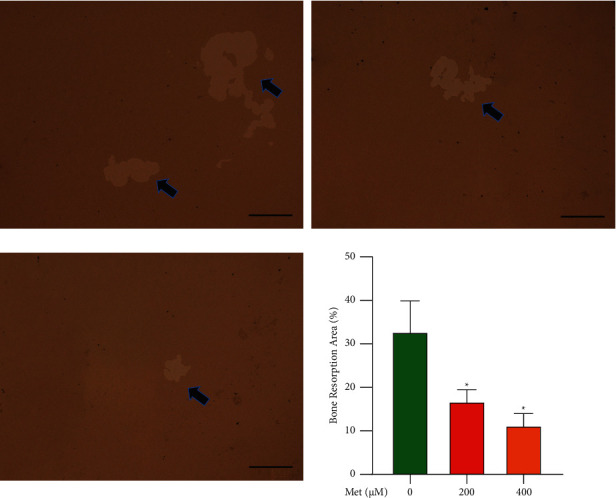
Met impaired bone resorption capacity of osteoclasts, BMMs were induced and cultured in medium with the addition of M-CSF (50 ng/mL), RANKL (100 ng/mL), and 0, 200, or 400 *μ*M Met for 5 d, and representative images of bone resorption area (%) were observed and photographed using a microscope (scar bar = 100 *μ*m, ^*∗*^*p* < 0.05). The black arrows indicate the bone resorption pits.

By cultivating BMMs in the wells of plates covered with hydroxyapatite, we were able to confirm the effect of Met on osteoclast bone resorption capabilities. The sizes of the resorption pits were assessed to evaluate the osteoclasts' resorption capacity. The resorption area was substantially lower in the Met-treated groups than in the Sham groups, and the impact was dose-dependent, demonstrating that Met inhibited osteoclast bone resorption capability (Figure 2).

### 3.3. Effect of Met on Osteoclastic F-Actin Ring Formation

**Figure 3:**
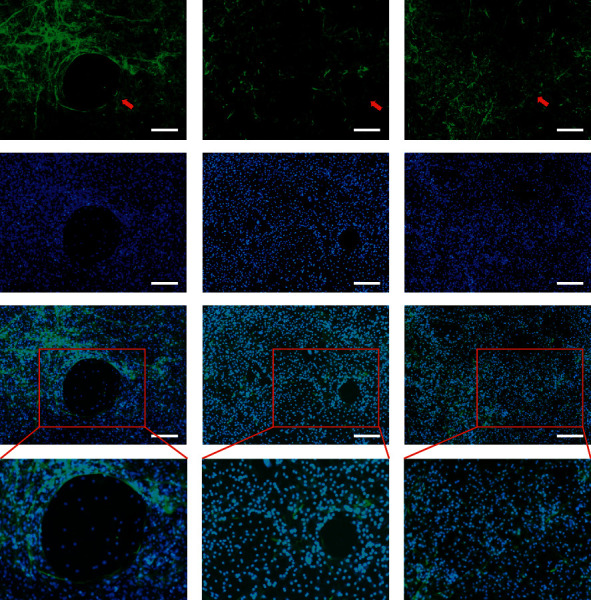
Met impaired osteoclastic formation of F-actin rings; BMMs induced and cultured in a medium with the addition of M-CSF (50 ng/mL), RANKL (100 ng/mL), and 0, 200, or 400 *μ*M Met for 5 d; then, the cells were stained with phalloidin and DAPI (scar bar = 100 *μ*m). The red arrows indicate the F-actin rings.

The actin cytoskeleton is a vital part of functional osteoclast maturation. Treatment with Met significantly decreased the size of the F-actin ring in RANKL-stimulated BMMs, supporting the results above (Figure 3).

### 3.4. Effect of Met on Osteoclastogenesis-Related Genes Expressed

**Figure 4:**
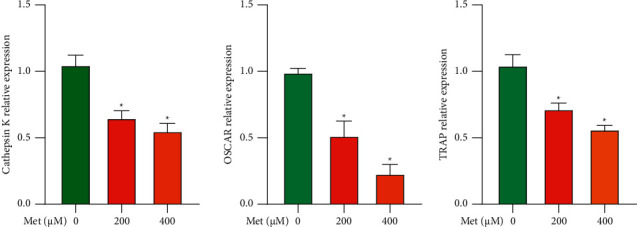
Met suppresses the expression of genes associated with osteoclastogenesis, such as *Cathepsin K*, *OSCAR*, and *TRAP*(^*∗*^*p* < 0.05). BMMs were induced and cultivated in media containing M-CSF (50 ng/mL), RANKL (100 ng/mL), and 0, 200, or 400 *μ*M Met for 5 d; then, the gene copies of *Cathepsin K*, *OSCAR*, and *TRAP* were quantified by real-time fluorescence quantitative PCR.

In this study, the effect of Met on osteoclast-related genes was examined by real-time fluorescence quantitative PCR. Different concentrations of Met (200 and 400 *μ*M) were used to interfere with osteoclast differentiation, and then, the expression of osteoclast-related genes (*Cathepsin K*, *OSCAR*, and *TRAP*) was examined. The results showed that Met inhibited osteoclast-specific genes (*Cathepsin K*, *OSCAR*, and *TRAP*) at 200 *μ*M and 400 *μ*M. This inhibition was concentration-dependent (Figure 4).

### 3.5. Effect of Met on ERK Phosphorylation

**Figure 5:**
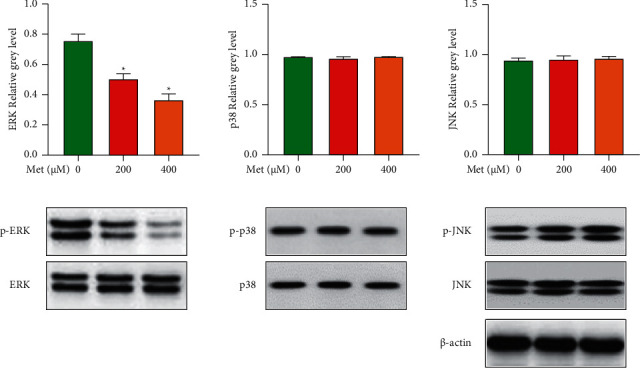
Met suppressed osteoclastogenesis by blocking the ERK signaling pathway while having a limited effect on the p38 or JNK signaling pathways. BMMs were induced and cultivated in media containing 50 ng/mL M-CSF, 100 ng/mL RANKL, and 0, 200, or 400 ng/mL Met for 5 d before being collected and lysed for Western blot analysis.

Met was used at different concentrations (200 and 400 *μ*M) to interfere with osteoclast differentiation, and then, the effect of Met on the MAPK signaling pathway was examined. According to the findings by collecting the cell proteins of each group after 5 d of Met treatment, Met had a limited effect on the activation of the MAPK subfamilies JNK and p38, according to the findings. The degree of phosphorylated ERK in the Met-treated group was much lower, and the impact was concentration-dependent, indicating that Met may block ERK phosphorylation (Figure 5).

## 4. Discussion

With the aging of the world population, osteoporosis has become an important disease affecting the quality of life, health, and safety of life of the global population. In diabetic patients, osteoporosis was first reported in 1948 and was named diabetoporosis in 2015 [9]. The current clinical treatment of osteoporosis is with drugs such as bisphosphonates, which are highly irritating to the gastrointestinal tract; parathyroid hormone requires daily subcutaneous injections and is expensive; calcium supplementation and vitamin D3 are not effective for osteoporosis. Therefore, the search for effective antiosteoporosis drugs with few side effects and long-term use is important to improve the quality of life of patients with osteoporosis.

Met is currently the first-line drug for the treatment of diabetes mellitus, with high efficiency and low side effects. Current literature reports that Met has anti-inflammatory, antitumor, hypolipidemic, and cardioprotective effects in addition to hypoglycemic effects [10]. It is well known that type II diabetic patients under Met treatment improve their BMD and improve indications of bone turnover markers in several international cohorts, including lowering serum adiponectin levels in patients [11, 12]. Met has also recently been found to inhibit osteoporosis, increase BMD, and reduce fracture risk in both diabetic osteoporosis model rats and diabetic osteoporosis patients [13, 14]. Current studies have shown that Met increases bone density by promoting osteoblast differentiation and enhancing the function of osteoblasts [6–8]; however, the effect of Met on osteoclasts is rarely reported in the literature.

Bone tissue is in a dynamic balance between osteoblasts leading to bone formation and osteoclasts leading to bone resorption. OPG, RANKL, and RANK are the three main cytokines that couple the differentiation and activation of osteoblasts, and osteoclasts play a very important regulatory role in the process of bone metabolism [15]. Osteoclasts are multinucleated cells of hematopoietic origin and are the main bone-resorbing cells. These cells are the main mediators of bone destruction. In the present study, we sought to establish a concentration-dependent effect of coadministration of Met and RANKL on osteoclast differentiation. We found that Met inhibited osteoclast bone resorption in a dose-dependent manner, significantly inhibiting the early stages of osteoclast differentiation and the formation of F-actin rings, which is a key function of osteoclasts [2]. Overall, these results indicate that Met not only inhibits osteoclast differentiation but also inhibits the overall function of osteoclasts. In addition, we used real-time fluorescence quantitative PCR to analyze and quantify RANKL-induced mRNA expression of osteoclast-related genes, including *Cathepsin K*, *OSCAR,* and *TRAP*. The mRNA levels of these genes were elevated after stimulation with RANKL for 5 days, whereas simultaneous treatment with Met inhibited this increase in the *Cathepsin K*, *OSCAR,* and *TRAP*.

We next investigate the underlying mechanisms of Met in osteoclast formation by Western blotting. Met inhibited the RANKL-induced ERK signaling pathway but had a very limited effect on p38 or the JNK signaling pathway, according to our data. MAPKs are a group of serine-threonine protein kinases that regulate inflammatory and innate immune responses [16]. The MAPK cascade is initiated by the phosphorylation of p38, ERK, and JNK in response to inflammatory signals [17]. Met suppressed cell survival while activating ERK signaling in human Ph + acute B lymphoblastic leukemia cells, according to Shi's work [18]. Met had a very limited effect on initiating autophagy and lowered ERK activity in HCT 15 human colorectal cancer cells, according to another investigation [19]. Met-induced ERK inhibition might be due to negative regulation between the RAS/RAF/MEK/ERK and PI3K/AKT/mTOR pathways, in which the activated PI3K/AKT/mTOR pathway suppressed ERK activity. Thus, greater research into how Met affects illnesses in various pathologies via ERK and other signaling pathways was required. The ERK signaling pathway has been implicated in the initiation of wear debris-stimulated osteolysis and osteoclastogenesis in previous studies [15, 20]. ERK is activated by the Ras-Raf-MEK1/2 pathway through MEK-mediated bifunctional threonine and tyrosine phosphorylation. ERK plays an important role in the formation, survival, differentiation, and stimulation of bone resorption of osteoclasts [21]. RANKL is a key regulator for activating the MAPK superfamily, containing ERK, which is phosphorylated preferentially by MEK1/2. After that, phosphorylated ERK stimulates c-Fos activation. This factor is a component of AP-1, which is an important translation factor in osteoclastogenesis. NFATc1 induction and translocation are also dependent on c-Fos. NFATc1 is a key transcription factor that is required for osteoclastogenesis and osteoclast activation, according to previous research [22]. Recent studies have shown that the MAPK signaling transduction pathway is closely related to bone metabolism [23]; ERK is the extracellular end point of various pro-proliferation signal transduction pathways and its core component, which can regulate the activity of osteoclasts. In this study, Met decreased the expression of osteoclastogenesis-related genes such as *TRAP*, *CTSK*, and *OSCAR* in a dose-dependent way. These impressive findings imply that Met suppresses phosphorylation of the ERK signaling pathway during osteoclastogenesis while having a limited effect on p38 and JNK signaling pathway activation.

Despite these intriguing results, our research has certain limitations. First, bone metabolism is a delicate balance between osteoclastic bone loss and osteoblastic bone production [24]. Bone production is also crucial in the treatment of osteolysis [25]. We have not investigated whether Met can improve osteoblast development in our model yet. This will be explored further in our future research. Second, the ovariectomized (OVX) model is an artificially induced ovarian function defect replicated in vivo model, first established by Saville in 1969 and then repeatedly validated and applied, which has become a classic model for the study of osteoporosis [26]. This model will be evaluated in our future studies for its ability to resist bone loss in Met in vivo.

**Figure 6:**
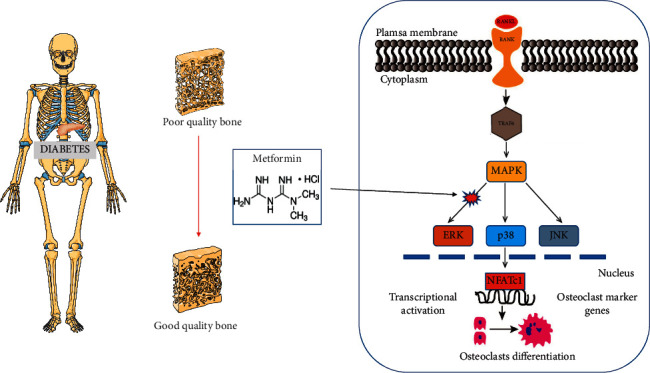
Graphical abstract. The present study demonstrates that Met inhibited osteoclastogenesis and the expression of osteoclastogenesis-related genes mainly via suppressing the ERK signaling pathway. These findings suggest that Met might be an effective therapy for osteoporosis.

In conclusion, the present study demonstrated that Met suppressed the ERK signaling pathway, which decreased osteoclastogenesis and the expression of osteoclastogenesis-related genes. This study suggests that Met might be an effective therapy for osteoporosis (Figure 6).

## Data Availability

The data used to support the findings of this study are available from the corresponding author upon request.
